# Fetal Growth Trajectories and Their Association with Maternal, Cord Blood, and 5-year Child Adipokines

**DOI:** 10.1155/2020/4861523

**Published:** 2020-09-23

**Authors:** H. C. Bartels, A. A. Geraghty, E. C. O'Brien, A. Kranidi, J. Mehegan, C. Yelverton, C. M. McDonnell, F. M. McAuliffe

**Affiliations:** ^1^UCD Perinatal Research Centre, School of Medicine, University College Dublin, National Maternity Hospital, Dublin, Ireland; ^2^Centre for Support and Training in Analysis and Research and School of Public Health, Physiotherapy and Sports Science, University College Dublin, Dublin, Ireland; ^3^Department of Paediatric Endocrinology & Diabetes, Children's Health Ireland, Temple Street Hospital, Dublin, Ireland

## Abstract

**Background:**

The growth of the fetus is a complex process influenced by multiple factors. Studies have highlighted the important role of biochemical growth markers such as leptin and adiponectin on fetal growth.

**Objective:**

To compare fetal growth trajectories with biochemical growth markers from maternal blood samples at 28 weeks' gestation, cord blood samples at birth, and in child blood samples at 5 years of age from mother-infant pairs who were part of the longitudinal ROLO study.

**Methods:**

781 mother-infant pairs from the ROLO and ROLO Kids study were included. Ultrasound measurements and birth weight were used to develop fetal growth trajectory groups for estimated abdominal circumference and estimated weight. Blood serum levels of leptin, adiponectin, insulin, TNF-alpha, and IL-6 from maternal, cord, and 5-year child samples were recorded. ANOVA and chi-square tests were applied to test the associations between fetal growth trajectory membership and maternal and child biochemical growth indicators. The influence of child sex was also investigated.

**Results:**

Male sex was associated with a faster weight trajectory compared to females (*p*=0.001). At 28 weeks' gestation, maternal leptin levels were significantly higher in mothers with a fetus on a slower estimated abdominal circumference trajectory compared to fast (25616 [IQR: 11656.0 to 35341.0] vs. 14753.8 [IQR: 8565.4 to 24308.1], *p* < 0.001) and maternal adiponectin levels were lower in fetuses on a slower estimated abdominal circumference trajectory compared to a fast trajectory (22.4 [IQR: 13.6 to 35.9] vs. 27.6 [IQR: 17.6 to 46.3], *p*=0.027). No associations were noted with inflammatory markers. No associations were identified between fetal growth trajectories and growth markers at 5 years of age.

**Conclusions:**

This study shows that male sex is associated with an accelerated estimated weight trajectory. Furthermore, high leptin and low adiponectin in maternal serum in late gestation are associated with a slower fetal growth trajectory. No associations were identified with blood growth markers after pregnancy.

## 1. Introduction

The growth of the fetus is a complex process influenced by multiple factors. Fetal growth is largely dependent on the supply of nutrients through the placenta [1]. The role of metabolic markers such as leptin and adiponectin in this process has received increasing interest and there is now an abundance of evidence that maternal nutritional status and body mass index influence fetal growth [2].

The role of adipose tissue as an endocrine organ that regulates metabolism and energy homeostasis and not simply as a storage depot for lipids is now well established [3]. Adipose tissue releases a large number of bioactive proteins into the circulation which have been collectively referred to as adipokines [4]. These include leptin and adiponectin. Maternal production of these adipokines and their role in fetal development and neonatal adipose concentration are key to understanding the underlying causes of fetal macrosomia. Delivery of nutrients to the fetus is a complex process regulated by insulin signaling and interaction with various cytokines, such as Tumor Necrosis Factor-alpha (TNF-alpha) and Interleukin 6 (IL-6) [5]. Maternal obesity has been associated with accelerated fetal growth and macrosomia and animal models have demonstrated accelerated placental nutrient transfer in obese mice [6, 7].

Leptin plays an important role in regulating energy homeostasis, metabolism, and appetite control by communicating nutritional status to neuronal targets in the brain [8]. Circulating levels of leptin are directly related to the amount of body fat and hence reflect the individuals' energy stores [9]. Leptin is secreted throughout pregnancy both by maternal adipose tissue and directly from the placenta, with the highest levels produced in the third trimester [10]. Previous studies have found an association with leptin levels at birth and BMI *z* score in childhood, with a positive correlation between high leptin levels at birth and high BMI *z* scores at 8 years of life [11]. Further studies have found low leptin levels at birth associated with lower birth weight, but an initial increased weight gain in the first 6 months of life [12]. Low levels of leptin are associated with growth restricted fetuses compared to well grown controls [13]. Furthermore, maternal leptin levels in early pregnancy are related to fetal size at 34 weeks and birth weight [14]. Differences in leptin levels according to fetal sex are also present, with cord leptin levels lower in males compared to females [15].

Adiponectin is the most abundant peptide produced by adipocytes and has a number of functions, in particular relating to insulin sensitization and regulating anti-inflammation [16]. Low levels of adiponectin are associated with cardiovascular disease, obesity, and type 2 diabetes, reflecting the importance of adequate adiponectin in regulating insulin and atherosclerosis [17]. Adiponectin has various end-organ targets, including skeletal muscle, where it promotes glucose uptake in cells [18], the endothelial vasculature, where adiponectin is a significant cardio-protector [19], and adipose tissue itself where overexpression has been found to protect against the lipotoxic effects of lipid accumulation related to the consumption of high-fat diets [20]. Adiponectin is not directly produced by the placenta and its influence on fetal growth is due to maternal adipose tissue production [21]. Pregnant patients with obesity typically have low levels of adiponectin and this may contribute to excessive fetal growth and macrosomia [22]. Several studies have demonstrated a negative correlation with maternal serum adiponectin and birth weight, with the highest birth weights associated with the lowest levels of adiponectin [23, 24]. This finding has been confirmed in both pregnant patients with gestational diabetes (GDM) and healthy pregnant women [24, 25]. Furthermore, placentas of patients with macrosomic infants have been found to have lower expression of adiponectin receptors, and patients with large-for-gestational age infants have a more rapid decrease in adiponectin levels as gestation advances compared to controls with normal-sized infants [26]. Replacement of adiponectin in obese pregnant mice was found to reverse insulin resistance and normalize fetal growth [27].

Maternal insulin plays an important role in fetal growth, and disturbances in insulin levels have been found to influence fetal growth, with excessive insulin leading to fetal macrosomia [28]. Insulin does not cross the placenta and influences fetal growth by acting on insulin receptors on the placenta [29]. Pregnancies complicated by diabetes have been found to have insulin receptor defects, hence resulting in a defect in the insulin signaling pathway [30].

TNF-alpha and IL-6 are proinflammatory cytokines produced by macrophages during acute inflammation and play an important role in cell signaling, leading to necrosis or apoptosis [31]. The placenta produces TNF-alpha and IL-6 throughout pregnancy [32]. TNF-alpha plays a key role in allowing implantation and trophoblast development, with levels increasing with advancing gestation as the placenta grows [33]. A number of studies have compared cord levels of TNF-alpha and IL-6 in growth restricted fetuses to well grown controls and found significant elevations in TNF-alpha and IL-6 [34, 35]. Both TNF-alpha and IL-6 stimulate placental amino acid transport and Il-6 also upregulates fatty acid uptake in trophoblast cells [36].

Studies on these growth biomarkers to date have been cross-sectional, focusing on one time point and analysis on longitudinal samples and growth patterns are limited. The purpose of this study is to explore the role of fetal sex and the adipokines leptin and adiponectin, insulin, and the cytokines TNF-alpha and IL-6 in fetal and child blood and to assess their association, if any, with fetal growth trajectories.

## 2. Methods

This is a secondary analysis of data from the ROLO and ROLO Kids studies. Detailed study methodology and findings have been previously published [37]. In brief, the ROLO study was a randomized control trial including 781 mother-infant pairs to compare the effect of a low GI diet to prevent recurrence of macrosomia. Women were recruited in early pregnancy at first antenatal consultation at a mean gestational age of 12.9 ± 3.0 weeks. As per the study protocol, all women were secundigravid having previously delivered an infant weighing greater than 4000 g. The intervention did not influence fetal weight and there was a moderate reduction in gestational weight gain and maternal glucose intolerance in the intervention group.

Data from the ROLO study was used to develop fetal growth trajectories, with methods to develop the trajectory models previously described in detail [38]. In summary, fetal measurements were obtained from ultrasound scans performed on mothers at medians of 20 + 6 (IQR: 20 + 1 to 21 + 5) and 34 + 1 (IQR: 33 + 5 to 34 + 5) weeks' gestation, including AC, head circumference, biparietal diameter, and femur length. Ultrasound measurements were performed using a Voluson 730 Expert (GE Medical Systems, Germany). An estimated fetal weight (EFW) at 20 and 34 weeks' gestation was calculated using the Hadlock 4-parameter formula. Two fetal trajectory types were identified: estimated abdominal circumference and estimated weight. The two estimated abdominal circumference growth trajectories comprised 29% of the participants on a slower trajectory and 71% on a fast trajectory [39]. For estimated weight, the four trajectories comprised 4% on a slow trajectory, 63% in a moderate trajectory, 30% in a moderate-fast trajectory, and 3% on a very fast trajectory.

As part of the ROLO and ROLO Kids study, longitudinal measurements of leptin, adiponectin, insulin, homeostatic model assessment of insulin resistance (HOMA-IR), TNF-alpha, and IL-6 concentrations have been collected.

For this study, fasting maternal blood samples collected at recruitment at 12.9 ± 3.0 and 28 weeks (leptin, adiponectin, insulin, HOMA IR, TNF-alpha, and IL-6), samples from cord blood (leptin, adiponectin, TNF-alpha, and Il-6), and nonfasting samples from children at 5 years of age (leptin and adiponectin) were compared with the aforementioned fetal growth trajectories. For maternal samples at 28 weeks' gestation leptin, adiponectin, insulin, HOMA-IR, TNF-alpha, and IL-6 were recorded. Plasma concentrations of leptin, insulin, and insulin were determined by the Human Endocrine Panel. HOMA-IR was calculated as (fasting insulin *μ*U/mL *x* fasting glucose mmol/L)/22.5 [40]. For cord blood, 108 samples of leptin, adiponectin, TNF-alpha, and IL-6 were recorded and, at the 5-year time point, 83 serum levels of leptin and adiponectin were available for analysis.

There were no differences found in the levels of any biochemical markers between the intervention and control group in the original ROLO study; hence the data for the trajectory models was pooled.

Statistical analysis was performed using SPSS Windows version 24.0 (SPSS Version 24, IBM). Nonnormally distributed data was analyzed using parametric testing, Mann–Whitney, and Kruskal–Wallis, with post hoc corrections for multiple measurements by the Holm–Bonferroni method. Chi-square was used to compare categorical variables. Statistical significance was set at *p* < 0.05.

Ethical approval was granted by the National Maternity Hospital Ethics Committee (June 2007).

## 3. Results

Characteristics of the cohort are described in Table 1. Participants were predominantly of white Irish ethnicity and, at delivery, were aged 32 years on average, with the mean BMI in the overweight range (27 kg/m^2^). As per the ROLO trial protocol, mothers with comorbidities were excluded.

### 3.1. Child Sex

**Figure 1:**
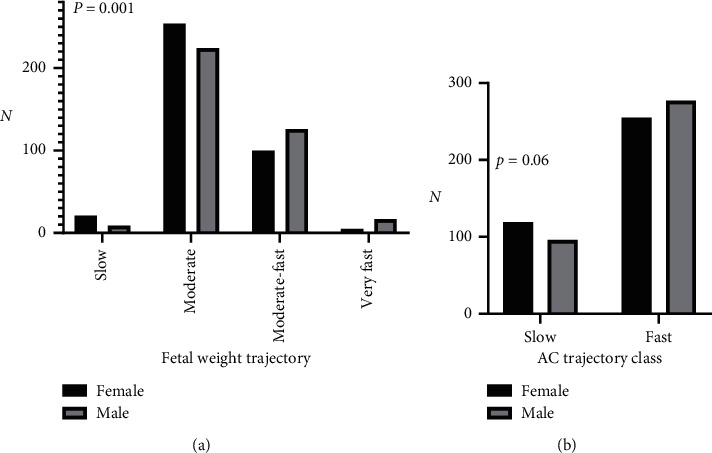
(a) Fetal growth trajectory (slow, moderate, moderate-fast, very fast) and association with fetal sex. (b) Abdominal circumference (slower and fast) and association with fetal sex.

Comparing the growth trajectory of male and female fetuses, males were found to grow on a faster trajectory compared to females for the estimated weight trajectory (Figure 1(a)). For the estimated abdominal circumference trajectory, there was a trend towards a faster trajectory for males but this did not reach statistical significance (*p*=0.06, Figure 1(b)) (Table 2). In line with this, at birth, female infants were significantly lighter compared to male infants (3945 ± 436 vs. 4081 ± 549 g, *p* < 0.001). There was no difference between males and females in associations of any biochemical markers with fetal estimated abdominal circumference or estimated weight growth trajectories.

### 3.2. Maternal Samples

**Figure 2:**
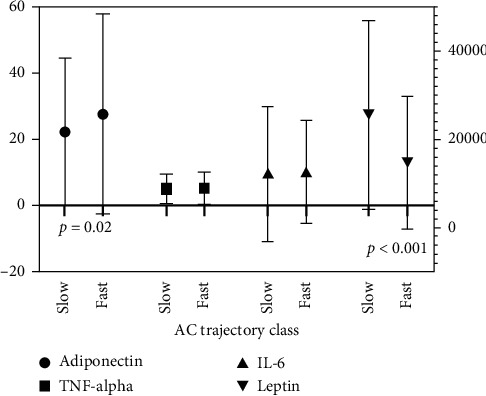
Maternal 28-week samples and association with abdominal circumference trajectory (slower and fast).

Maternal samples at 28 weeks' gestation were recorded for leptin, adiponectin, insulin, HOMA IR, TNF-alpha, and IL-6. For the estimated abdominal circumference trajectory, maternal leptin levels were significantly higher in a fetus on a slower trajectory compared to a fast trajectory (25616 ng/ml [IQR: 11656.0 to 35341.0] vs. 14753.8 ng/ml [IQR: 8565.4 to 24308.1], *p* < 0.001) (Figure 2). Maternal adiponectin levels were lower in fetuses on a slower estimated abdominal circumference trajectory compared to a fast trajectory (22.4 ng/ml [IQR: 13.6 to 35.9] vs. 27.6 ng/ml [IQR: 17.6 to 46.3], *p*=0.027) (Figure 2). No significant differences in levels of TNF-alpha or IL-6 were found for the estimated abdominal circumference trajectory. For the estimated weight, there was no difference in levels of leptin, adiponectin, HOMA-IR, or insulin in mothers with a fetus on a slower or faster estimated weight trajectory (Table 3). Maternal samples in early pregnancy (12.9 ± 3.0) were also assessed for association with fetal growth trajectories and no significant differences in biomarkers were found.

### 3.3. Cord Blood Samples

105 cord samples were available for analysis, from which cord leptin, adiponectin, TNF-alpha, and IL-6 were measured. For the estimated abdominal circumference and the estimated weight trajectory, there was no difference in levels of cord leptin or adiponectin (Tables 3 and 4). There was no difference in cord TNF-alpha and IL-6 between the fetal estimated abdominal circumference or the estimated weight trajectories.

### 3.4. Child Samples at 5 Years

At 5 years of age, 83 children had blood samples taken as part of follow-up for the ROLO Kids study. There was no association between the fetal estimated abdominal circumference or estimated weight groups and leptin and adiponectin concentrations at 5 years of age. There was no difference in biomarker concentrations between male and female children and child sex did not impact associations between the biomarkers and estimated abdominal circumference and estimated weight (Table 4).

## 4. Discussion

The main findings of this study are an accelerated growth trajectory in male compared to female fetuses and an association between maternal levels of leptin and adiponectin in late pregnancy and fetal growth trajectories, but not in cord blood or later child blood.

This study shows a significant difference in the growth trajectory for males compared to females for the estimated weight trajectory, with male fetuses growing on a faster trajectory compared to females. Sex differences in the fetal growth rate were first described in 1963 [41]. The underlying mechanisms are multifactorial, with evidence suggesting epigenetic differences between male and female embryos leading to changes in the speed of development of growth and metabolism [42]. In vitro cultured male embryos have a higher metabolic rate and grow faster compared to female embryos, which likely translates into faster growth in utero [43]. While the levels of placental growth hormone at 28 weeks have been found to be higher in women carrying a female fetus, this was not found to correlate with birth weight, suggesting determinants of fetal growth are established early in pregnancy [44]. A previous study found higher levels of IGF-1 and insulin in cord samples of females compared to males [45]. Anthropometric data from neonates shows a higher ratio of subcutaneous fat in females compared to males, with evidence suggesting male fetuses gain more weight but lose more fat in the last few weeks of pregnancy. This study adds further weight to that theory, as male fetuses were found to grow on a faster trajectory with a corresponding significantly higher birth weight.

The accelerated growth of male fetuses starts from the first trimester, with male fetuses having a longer crown-rump length compared to females, with persistent larger ultrasonographic measurements of AC as gestation advances in males [46, 47]. A study of nutritionally at-risk women found differences in the birth weight of males and females following nutritional supplementation during pregnancy with males gaining more weight than females in the treatment group [48]. Male fetuses exposed to GDM were at increased risk of having a BMI in the obese range at 5–7 years while no such effect was seen in females [49]. The phenomenon of “male vulnerability,” where male neonates have higher mortality rates and morbidity including intraventricular hemorrhage and respiratory distress syndrome is well established; however, the underlying mechanisms are poorly understood [50]. Hence, there are clear differences in the growth and responses to in utero exposures in males and female fetuses. However, the underlying mechanisms are not clear. Further evidence is needed to determine if this process is placental or fetal driven, whether the XY placenta allows greater substrate transfer to the fetus or if a male fetus demands more nutrients in utero.

Previous work by our group found higher levels of cord leptin in female fetuses compared to males [51], however, we found no differences in fetal sex and associations with biochemical markers and fetal growth trajectory grouping. Furthermore, we previously found that fetal sex did not impact maternal and fetal metabolic parameters in women with a BMI > 25 kg/m^2^ [52].

This study also found that maternal levels of leptin and adiponectin are associated with fetal growth patterns, with higher levels of leptin and lower levels of adiponectin found at 28 weeks in mothers with a fetus on a slower estimated abdominal circumference trajectory. This is in contrast with the previous data, where higher levels of leptin have been associated with higher birth weight and BMI [14] and the highest birth weights associated with the lowest levels of adiponectin [23, 24]. Previous studies have found lower levels of adiponectin in obese women compared to lean women throughout pregnancy [5, 23], and adiponectin levels were inversely related to fetal growth [53]. At a placental level, adiponectin causes insulin resistance [54] by activating PPAR*α* and inhibiting insulin receptor substrate 1 phosphorylation, which reduces insulin responsiveness [55]. Hence, when adiponectin levels are low, placental insulin response is not limited appropriately and fetal growth accelerates. However, we found that lower levels of adiponectin were seen in the maternal blood of the slower growing fetus. In the third trimester of pregnancy, leptin upregulates placental System A amino acid transport, which results in increased nutrient uptake by the fetus [56]. Hence, higher leptin levels in obese mothers may play a role in accelerated fetal growth as a result of increased nutrient supply. Again, our results differed in that we found leptin levels were lower for the mothers with a fetus on a higher trajectory. One hypothesis is that the faster growing fetus consumes more leptin to accelerate growth, hence accounting for the lower levels in the faster growing cohort. Alternatively, the maternal environment may be trying to encourage growth in the slower growing fetus by increasing leptin and decreasing adiponectin. Interestingly, we did not find any differences between male and female biomarkers and growth trajectory. Furthermore, HOMA-IR and insulin levels in maternal serum levels were similar. Hence, the underlying mechanism to explain the association between high leptin and low adiponectin and a slower growth trajectory is unclear.

This study also measured Inflammatory markers to compare their association with fetal growth trajectory and offspring sex as our group had previously found an association amongst females with inflammatory factors and infant adiposity [57]; however, this was not seen in this growth trajectory analysis.

For cord blood levels, no biochemical markers were significantly associated with fetal growth. No other significant associations were found with levels of adiponectin or TNF-alpha and the fetal growth trajectories. This reflects the complex fetal growth process which is influenced by multiple factors. While previous studies have found an association with high cord levels of leptin and increased birth weight, including a previous analysis of data from this cohort [14], we did not find such an association with the growth trajectory of the fetus. Hence, while crude birth weight may be associated with increased leptin levels, the actual trajectory of growth may not be related to maternal or cord leptin levels.

The main strength of this study is the large number of samples collected at longitudinal time points including maternal, cord, and child samples. The data was collected as part of a well-designed randomized control trial. To our knowledge, this is the first study to compare biochemical growth makers with fetal growth trajectories. This study is limited by a number of considerations. Firstly, all mothers included in this study had previously given birth to an infant weighing over 4000 g and the offspring were also, on average, in this range. Therefore, the reported trajectories may not be representative of all pregnant and infant populations. Furthermore, at 5 years the follow-up rate was 53.1% and hence there may be differences between those who attended for follow-up leading to potential influence on the results reported here. Of note, this figure is similar to the follow-up rates reported in the literature [58] and retention strategies were employed to attempt to improve the follow-up rate [59]. Greater differences in adipocytokines may have been seen in those with restricted intrauterine growth as has been shown in lower socioeconomic groups with accelerated postnatal catch-up growth [60]. This study is limited by the methods used to develop fetal growth trajectories. These are exploratory trajectories using novel methods that have not been reproduced and validated in other studies. Furthermore, the number of samples for analysis reduces significantly when split for trajectory class and gender and this reduces the power of the study. The child blood samples were nonfasting levels with a wide reference range and hence further work is required before we can ascertain if there is a longitudinal effect on adipocytokines from the antenatal environment.

In conclusion, we present novel data comparing adipokines with fetal growth trajectories. We found that male fetuses had a faster growth trajectory and that there was an association between leptin and adiponectin and fetal growth. This data adds to our understanding of the complex interactions between maternal adipokines, fetal sex, and growth trajectories.

## Figures and Tables

**Table 1:** ROLO Demographics—baseline characteristics of total cohort.

Maternal characteristics (*n* = 781)	
Age (years)	32.4 (4.2)
Weight (kg)	73.5 (14.0)
Height (cm)	166.0 (6.4)
Body mass index (kg/m^2^)	26.7 (4.9)
Fasting glucose at booking (mmol/L)	4.5 (0.57)
Glucose intolerance^*∗*^, *N* (%)	184 (25%)
Smoking during pregnancy, *N* (%)	30 (4%)

Child characteristics	
Male sex, *N* (%)	376 (50%)
RCT intervention group, *N* (%)	369 (49%)
Gestational age (weeks + days)	40 + 2 (1 + 1)
Birth weight (kg)	4.03 (0.48)
Birth weight centile	72 (25)
5-year weight (kg)	20.3 (2.6)
5-year BMI centile	63 (25)

Maternal characteristics at study enrollment (approx. 14 weeks' gestation). ^*∗*^Glucose intolerance at 28 weeks' gestation as defined by fasting glucose > fasting glucose ≥5.1 mmol/L or glucose challenge test (50 g glucose load) > 7.8 mmol/L. Values are mean (SD) unless stated otherwise.

**Table 2:** Abdominal circumference and estimated weight trajectory class and child sex figures in number with (%).

Abdominal circumference trajectory class	Female (*n* = 374)	Male (*n* = 373)	*p* value^*∗*^

Slower (*n*, %)	119 (32)	96 (25.7)	0.067
Fast (*n*, %)	255 (68)	277 (74.3)	

Weight trajectory class	Female (*n* = 380)	Male (*n* = 376)	*p* value

Slow (*n*, %)	21 (5.5)	9 (2.5)	0.001
Moderate (*n*, %)	254 (66.8)	224 (59.5)	
Moderate-fast (*n*, %)	100 (26.4)	126 (33.5)	
Very fast (*n*, %)	5 (1.3)	17 (4.5)	

^*∗*^Chi-square.

**Table 3:** Estimated fetal weight trajectory class (slow, moderate, moderate-fast, very fast) and association with biochemical markers.

Weight trajectory class	Total			Slow (4%)			Moderate (63%)			Moderate-fast (30%)			Very fast (3%)			*p* value^*∗*^
	*n*	Median	IQR	*n*	Median	IQR	*n*	Median	IQR	*n*	Median	IQR	*n*	Median	IQR	
Maternal samples at 28 weeks																
Leptin (pg/ml)	531	19641.7	(14518.6, 34071.8)	23	15578.8	(5815.9, 25562.5)	330	15711.1	(8739.2, 26297.5)	162	16270.9	(8876.2, 24449.0)	16	18891.8	(13684.1, 31437.9)	0.784
Adiponectin (ng/ml)	291	24.1	(16.6, 37.8)	12	22.7	(16.1, 26.6)	185	26.1	(15.1, 42.4)	87	27.9	(15.9, 50.3)	7	17.6	(15.2, 31.1)	0.315
HOMA-IR	507	3.1	(1.7, 5.1)	21	3.21	(1.8, 5.8)	316	2.8	(1.6, 4.3)	155	3.1	(1.8, 5.4)	15	2.1	(1.2, 5.1)	0.567
Insulin (IU)	531	17.2	(9.0, 25.6)	22	16.5	(9.5, 29.1)	334	14.3	(8.9, 22.1)	160	15.3	(8.8, 26.5)	15	9.8	(6.4, 26.5)	0.302

Cord blood samples																
Cord leptin (ng/ml)	108	28479.9	(16482.7, 47203.2)	3	11765.3	(10556.3, 22808.5)	75	19633.1	(12751.4, 35212.8)	27	22162.9	(8945.7, 42044.6)	3	19288.0	(12621.2, 37457.4)	0.212
Cord adiponectin (ng/ml)	108	80.2	(22.0, 915.1)	3	96.2	(37.7, 192.4)	75	141.3	(62.5, 327.5)	27	139.4	(46.7, 227.7)	3	57.9	(43.7, 78.5)	0.700

5-year child samples																
Leptin (ng/ml)	83	77.0	(19.7, 151.6)	1	443.1	-	54	184.6	(87.8, 418.1)	24	242.6	(74.8, 465.9)	4	30.2	(13.1 -	0.880
Adiponectin (ng/ml)	83	17.1	(7.5, 21, 7)	1	0.2	-	54	11.4	(4.2, 17.9)	24	13.9	(5.6, 18.5)	4	11.6	1.6 -	0.913

^*∗*^Kruskal–Wallis.

**Table 4:** Abdominal circumference trajectory (slower or fast) associated with biochemical markers.

Abdominal circumference trajectory class	Total			Slower (29%)			Fast (71%)			*p* value^*∗*^
	*n*	Median	IQR	*n*	Median	IQR	*n*	Median	IQR	
Maternal blood at 28 weeks										
Leptin (ng/ml)	524	16970.1	(9705.2, 26962.5)	129	25616.4	(11656.0, 35341.0)	395	14753.8	(8565.4, 24308.1)	<0.001
Adiponectin (ng/ml)	290	25.6	(15.4, 41.8)	91	22.4	(13.6, 35.9)	199	27.6	(17.6, 46.3)	0.027
HOMA-IR	503	2.9	(1.5, 4.7)	129	3.2	(1.6, 5.2)	374	2.8	(1.5, 4.8)	0.258
Insulin (IU)	526	14.6	(8.0, 23.0)	134	15.7	(8.7, 26.0)	392	14.5	(8.0, 23.8)	0.258

Cord blood samples										
Cord leptin (ng/ml)	105	18567.1	(14477.7, 41437.6)	22	21072.4	(11795, 35439.4)	83	19503.9	(12015.7, 37501.2)	0.892
Cord adiponectin (ng/ml)	105	104.1	(22.0, 915.1)	22	144.0	(53.5, 270.4)	83	131.0	(59.1, 131.0)	0.877

5-year child samples										
Leptin (ng/ml)	83	77.0	(19.7, 151.6)	24	205.1	(70.8, 443.0)	59	169.8	337.3	0.791
Adiponectin (ng/ml)	83	17.1	(7.5, 21.7)	24	13.6	(0.8–18.1)	59	10.2	12.8	0.931

HOMA-IR: homeostatic model assessment of insulin resistance, ^*∗*^Mann–Whitney.

## Data Availability

All relevant data that support the findings of this study are included within the paper. Any additional data required may be requested from the corresponding author.
